# Supplementary material for: Single microtubules and small networks become significantly stiffer on short time-scales upon mechanical stimulation
Source: Sci Rep. 2017 Jun 26;7:4229. doi: 10.1038/s41598-017-04415-z (PMC5484680; doi:10.1038/s41598-017-04415-z)
Supplement: Supplementary file 1 — Supplementary Information [file 41598_2017_4415_MOESM1_ESM.pdf]

Single microtubules and small networks become significantly stiffer on short time-scales upon mechanical stimulation

Supplementary information

Matthias D. Koch[a,1], Natalie Schneider[b], Peter Nick[b], and Alexander Rohrbach[a]

[a] Laboratory for Bio- and Nano-Photonics, Department of Microsystems Engineering, University of Freiburg, Georges-Koehler-Allee 102, 79110 Freiburg, Germany

[b] Molecular Cell Biology, Botanical Institute, Karlsruhe Institute of Technology, Kaiserstr. 2, 76131 Karlsruhe, Germany

[1] Present address: Lewis-Sigler Institute for Integrative Genomics, Princeton University, Washington Rd, Princeton, NJ 08544, USA

SI Methods and Material

The elastic relaxation forces along the filament $\int dF_{\kappa,MT}$ are reduced by filament friction $\int dF_{\gamma,MT}$ and act in lateral y direction. Due to the homolog constraint of the filament of constant length L and its connection to the optically trapped beads, the resulting elastic forces of the microtubule $F_{\kappa,MT}$ push the beads outwards in x-direction and are counteracted by the optical forces F_{opt}. Whereas the friction force $F_{\gamma,B}$ on the bead in the sensor trap (blue) is negligible small, the viscous force on the oscillating actor bead (red) counteracts the driving force F_{drive}.

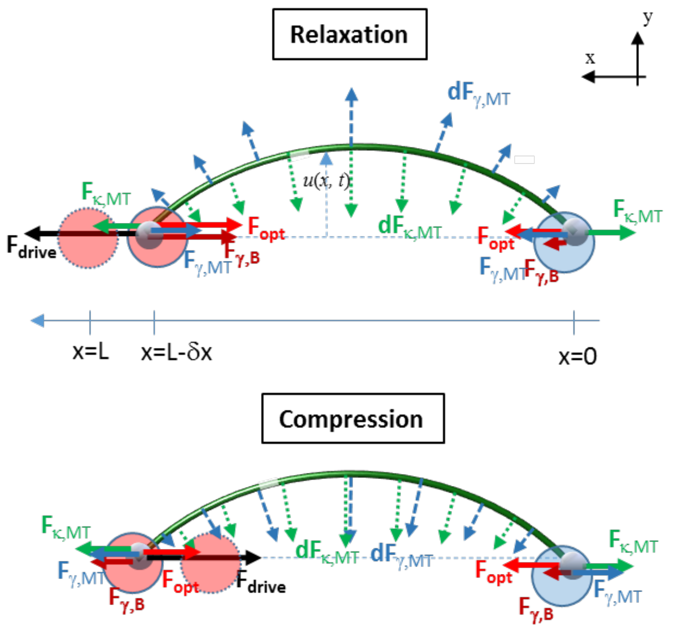

Fig. S1. Force diagrams of a buckling filament held by two optical traps in the case of relaxation or compression, for a pulling or a pushing driving force, respectively. The local elastic forces on different parts of the filament and the resulting elastic forces are shown in green, all viscous forces are shown in blue.

The tension free equation of motion for relaxation (F_{drive} in positive x-direction) reads

$$-F_{opt1}(x_{B1}) - F_{\gamma_{B1}}(x_{B1}) + \tfrac{1}{2}F_{x,\kappa_{MT}}(u(x)) - \tfrac{1}{2}F_{x,\gamma_{MT}}(u(x)) = -F_{drive}(x_{B1}) \quad \text{for the left bead and}$$

$$+F_{opt2}(x_{B2}) + \underbrace{F_{\gamma_{B2}}(x_{B2})}_{\to 0} - \tfrac{1}{2}F_{x,\kappa_{MT}}(u(x)) + \tfrac{1}{2}F_{x,\gamma_{MT}}(u(x)) = 0 \quad \text{for the right bead.}$$

Micro-rheology analysis

The measured, frequency dependent displacements x_{Bi}, y_{Bi} of bead i as a response to an applied actuation force $F_x^{(j)}$, $F_y^{(j)}$ on bead j is given by the response functions $A_x^{(i,j)}$, $A_y^{(i,j)}$ according to Eq. (S1)

$$\begin{aligned} x_{Bi}(\omega)\cdot\frac{1}{A_x^{(i,j)}(\omega)} &= F_x^{(j)}(\omega) \\ y_{Bi}(\omega)\cdot\frac{1}{A_y^{(i,j)}(\omega)} &= F_y^{(j)}(\omega) \end{aligned} \qquad \text{(S1)}$$

In active micro-rheology as it is used here, the driving force $F = \kappa{\cdot}x_L(t)$ is generate by a sinusoidal oscillation $x_L(t) = A_a\sin(\omega_a t)$ of one optical trap with stiffness κ, amplitude A_a and driving frequency ω_a. To obtain the complete spectrum $A(\omega)$, the experiment has to be repeated several times for different actuation frequency ω_a and evaluated according to Eq. (S1) for each frequency ω_a. As explained in the main paper, the measured bead displacements x_B, y_B are a superposition of the elastic trapping force, the viscous drag of beads and the wanted viscoelastic properties of the material under investigation, i.e., of the microtubule filaments in our case. Hence, the response function A is a superposition of these contributions as well. As explained in (1, 2) given by Eq. (S2), this can be separated to obtain the pure viscoelastic response function G_{MT} of the filament:

$$\begin{aligned} G_x^{(i,j)} &= \frac{1}{4\pi L\cdot A_x^{(i,j)}}\left(1-\kappa_{xi}A_x^{(i)} - \kappa_{xj}A_x^{(j)} + \kappa_{xi}\kappa_{xj}\left(A_x^{(i)}A_x^{(j)} - \left(A_x^{(i,j)}\right)^2\right)\right) \\ G_y^{(i,j)} &= \frac{1}{8\pi L\cdot A_y^{(i,j)}}\left(1-\kappa_{yi}A_y^{(i)} - \kappa_{yj}A_y^{(j)} + \kappa_{yi}\kappa_{yj}\left(A_y^{(i)}A_y^{(j)} - \left(A_y^{(i,j)}\right)^2\right)\right) \end{aligned} \qquad \text{(S2)}$$

Here, $\kappa^{(i)}$ and $\kappa^{(j)}$ are the trap stiffnesses of the corresponding traps which have to be determined independently by calibration (3, 4). Different pre-factors $4\pi L$ and $8\pi L$ for different directions take care of the hydrodynamic coupling $\gamma_{HC,\mathrm{y}} = 2\gamma_{HC,\mathrm{x}} = 8\pi L\eta\omega$ of distant sites (1) separated by the distance L. In the main paper, we usually used the notion parallel (||) and perpendicular ($\perp$) instead of x and y according to the direction of oscillation with respect to the filament orientation.

To ensure correct results we tested the software implementation and measurement procedure for simple beads in water, where the viscoelastic response is known theoretically and measured experimentally (1). Since the motivation of the paper is to study the transport of mechanical stimuli, only the elastic components $G'^{(i,j)}$ (real part of G) are shown and

discussed in the main paper. Viscous components (imaginary part of *G*) are shown in the SI Results (see below).

The molecular architecture of differently polymerized and stabilized filaments

We choose filaments polymerized in the presence of a non or slowly hydrolysable GTP analog (GMPCPP) in addition to filaments assembled with GTP since both filament types have significantly different mechanical properties (5) ultimately governed by different molecular configurations as illustrated in Fig. S2. After polymerization, GTP molecules in the microtubule lattice hydrolyze stochastically to GDP. While GTP and GTP-analog tubulins adopt a straight conformation, the hydrolysis at the β-tubulin leads to a kink of the GDP tubulin dimer resulting in an intrinsic strain in the microtubule lattice (6, 7). This conformational change is slowed down by Taxol (8) which binds on the inside of the hollow tube (9, 10) and has been used to theoretically recapitulate the tip structure and rates of assembly/disassembly of microtubules (11), the occurrence of long-lived arcs and rings in kinesin-driven gliding assays (12) and to transform MTs into inverted tubules facing their inside out by a specifically induced conformational change using spermine, a polyamine present in eukaryotic cells (13). Further, microtubules polymerized in the presence of slowly or non-hydrolyzable GTP analogs such as GMPCPP or γ-S-GTP have additional lateral inter-protofilament contacts between β-tubulins compared to GTP/GDP microtubules (5, 14). Assuming that the connection between individual αβ- tubulin dimers can be approximated by damped harmonic springs (15, 16), the damping of the intermolecular connections should affect the temporal response upon exertion of mechanical stimuli and thereby the transition frequency ω_t.

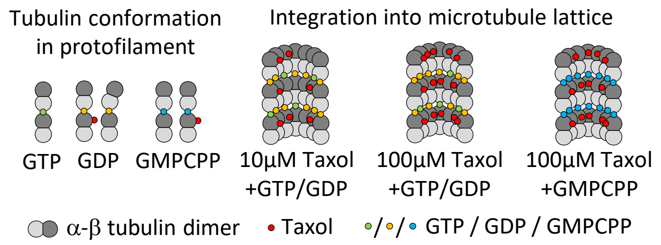

Fig. S2. Effect of GTP, GDP, GMPCPP, Taxol and combinations thereof, on molecular conformation of the αβ-tubulin dimer and corresponding binding sites.

SI Results

Lateral forces are negligible

In addition to the data shown in Fig. 2 of the main paper, we here compare the bead displacements along the x and y direction during a single filament rheology experiment at two oscillation frequencies f = 0.1Hz and f = 100Hz. As Fig. S **3** shows, the total contributions in lateral y-direction are negligibly small. Here, the lateral elastic MT buckling force $F_{\kappa MT,y}(x,x_{Bj})$ is increased (reduced) by the MT drag force $F_{\gamma MT,y}(x, x_{Bj})$ for deformation (relaxation). Both MT forces are equilibrated by the strong optical forces $F_{opt,y}(x_{Bj})$ and the weak viscous drag forces of the beads $F_{\gamma,y}(x_{Bj})$ in lateral direction. The sum of these forces is zero for all oscillation frequencies and phasings, i.e., $F_{opt,y}(x_{Bj}) + F_{\gamma,y}(x_{Bj}) + F_{\kappa MT,y}(x, x_{Bj}) + F_{\gamma MT,y}(x, x_{Bj}) \approx 0$. This situation is revealed in Fig. S **3**:

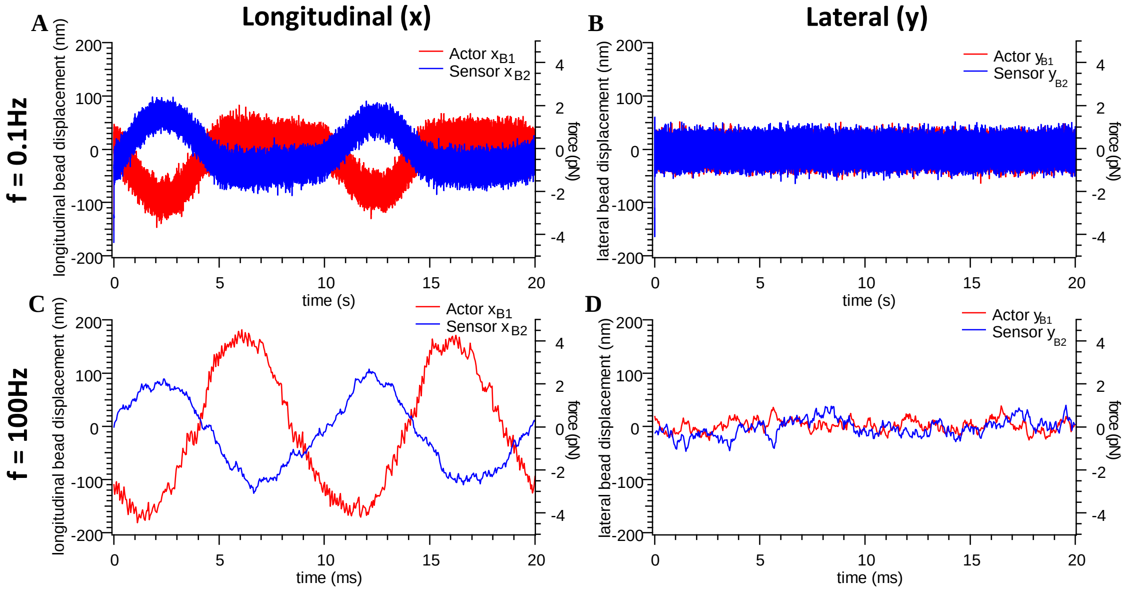

Fig. S 3. Responses of the actor and sensor bead resulting from the oscillatory driving of the actor bead in longitudinal (x) direction. Displacements and forces are large in longitudinal (x) direction (A and C), but very small in lateral (y) direction (B and D) for longitudinal oscillation frequencies at f = 0.1Hz (A and B) and f = 100Hz (C and D).

Frequency dependent bead displacements

The displacements x_{Bi} of the beads are governed by the elastic optical trapping force, the viscous drag force of the beads as well as the viscoelastic force from the microtubule filament according to Eq. (2) of the main article. In Fig. S4, we show the frequency dependence of the maximum actor and sensor bead displacement $|x_{Bi} - x_{Li}|$ during filament buckling and filament stretching. While an increase of the maximum amplitude of bead displacements of approximately one order of magnitude can be observed during buckling, bead displacements stay approximately constant and are proportional to the actor amplitude A_a during filament stretching. Already here, the connection between the constant low frequency plateau of G' and its power law rise above $f_t \approx$ 2Hz to filament buckling can be anticipated. The viscoelastic

contribution of the trapped beads alone moving in the purely viscous buffer medium is much smaller than the effect observed here and is dominated by the corner frequency $\omega_c = \kappa / 6\pi R_B \eta \approx 2500$Hz of the position power spectral density $|x_B(\omega)|^2$ of the bead motion, which is much larger than the transition frequencies estimated for our MT constructs.

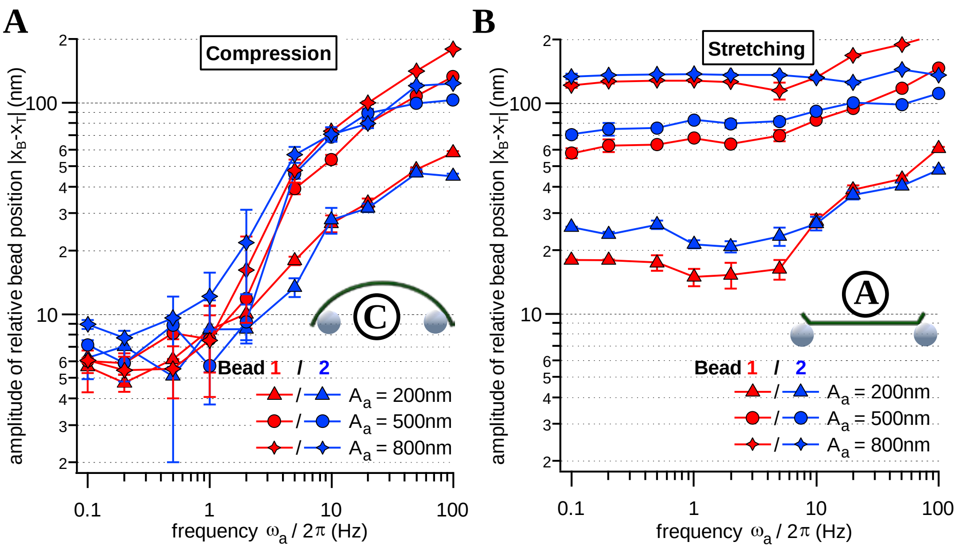

Fig. S4. Maximum displacement amplitudes $|x_{Bi} - x_{Li}|$ of both beads with index i relative to their trap centers x_{Li} during single filament oscillation. (A) During the compression (buckling) half period. (B) During the stretching half period.

Estimation of the total viscous force

To distinguish the absolute role of the different forces introduced in Eqs. (1) and (2) of the main paper, we determine these contributions in the following. The results shown here are the basis of Fig. 2D of the main paper.

The most obvious force is the viscous drag $F_{\gamma_B,\mathrm{tran}}$ of bead translation. The actor bead approximately follows the sinusoidal movement of its trapping focus and is much larger than that of the sensor bead, which is neglected for this reason. The movement of the actor trap is $x_{L1}(t) = A_a \sin(2\pi f_a t)$ resulting in the velocity $v_{L1}(t) = \partial/\partial t\, x_{L1}(t) = 2\pi f_a A_a \cos(2\pi f_a t)$. Only the maximal force components are considered in the following. Hence, the translational viscous drag force is given by Eq. (S3) and shown by the yellow line in Fig. S5B.

$$F_{\gamma_B,tran}(f_a) = \gamma_B \max(v_{L1}) = 12\pi^2 R_B \eta A_a f_a \qquad \text{(S3)}$$

The buckling filament causes both beads to rotate resulting in a rotational drag force $F_{\gamma_B,\mathrm{rot}}$ governed by a varying angle $\varphi_A(\delta_L) = \sqrt{\frac{4\delta_L}{L_{MT}}}$ for different compressions δ_L of the filament as illustrated in Fig. S5A. Beads are rotated at the angular velocity $\omega_{B,rot} = \Delta\varphi_A f_a$ with $\Delta\varphi_A = \varphi_A(A_a)$ resulting in the viscous torque $M = \gamma_{B,rot}\,\omega_{B,rot}$ with $\gamma_{B,rot} = 8\pi R_B \eta$ (17). This torque is

balanced by a viscous force according to $M = R_B F_{\gamma_B,\mathrm{tran}}$ resulting in Eq. (S4) and illustrated by green line in Fig. S5B.

$$F_{\gamma_B,rot}(f_a) = \frac{\gamma_{B,rot}\,\omega_{B,rot}}{R_B} = 8\pi\eta R_B^2 \sqrt{\frac{4A_a}{L_{MT}}}\, f_a \tag{S4}$$

To calculate the viscous drags $F_{\gamma_{MT},\parallel}(v_{\delta_L})$ and $F_{\gamma_{MT},\perp}(v_{\delta_L})$ acting on the MT during a parallel and perpendicular movement of the buckled filament with respect to the filament axis, the viscous drag coefficients of a rod $c_\parallel = \frac{2\pi\eta}{\ln(\frac{L}{D})-0.2}$ and $c_\perp = \frac{4\pi\eta}{\ln(\frac{L}{D})+0.84}$, the velocity of filament compression $v_{\delta L} = 2\pi f_a A_a$ and the buckling amplitude $u(\delta_L) = \sqrt{\left(\frac{L_{MT}}{\pi}\right)^2 - \left(\frac{L_{MT}-\delta_L}{\pi}\right)^2}$ as a function of filament compression δ_L is needed. The latter is deduced further below (see Eq. (S7)).

Assuming that the right side of the filament is approximately stationary while the left side moves at velocity $v_{\delta L}$, the parallel force component can be estimated by integrating the force per unit length of a small portion of the rod at position x and moving at a velocity $\frac{x}{L_{MT}-\delta_L} v_{\delta_L}$ over its entire length as illustrated in Fig. S5A. The result is shown in Eq. (S5) and Fig. S5B by the blue line.

$$F_{\gamma_{MT},\parallel}(f_a) = c_\parallel v_{\delta_L} \int_0^{L-\delta_L} \frac{x'}{L_{MT}-\delta_L} dx' = \frac{2\pi^2 \left(L_{MT}-A_a\right)\eta}{\ln\left(\frac{L_{MT}}{D}\right) - 0.2} A_a f_a \tag{S5}$$

In a similar fashion, the lateral force component can obtained by considering that both ends of the filament do not move in this direction in contrast to the filament center, and that the velocity of a small portion of the buckling rod at position x is determined by the temporal change $\frac{du(\delta_L)}{dt}\sin\left(\frac{\pi x}{L_{MT}-\delta_L}\right)$ of the buckling amplitude. This results in Eq. (S6) and is described by the black line in Fig. S5B.

$$F_{\gamma_{MT},\perp}(f_a) = c_\perp \frac{d}{dt} u(\delta_L) \int_0^{L-\delta_L} \sin\left(\frac{\pi x'}{L_{MT}-\delta_L}\right) dx' = \frac{16\left(L_{MT}-A_a\right)^2}{\sqrt{L_{MT}^2 - \left(L_{MT}-A_a\right)^2}} \frac{\eta A_a f_a}{\ln\left(\frac{L_{MT}}{D}\right) + 0.84} \tag{S6}$$

For comparison, we also plotted the experimentally obtained frequency dependence of the total force of a bead alone (yellow markers) and of a bead / MT construct (red markers) together with the total force of all contributions estimated above (red line) in Fig. S5B for an oscillation amplitude A_a = 600nm. For the situation of a bead alone, the mean force $F \approx 0.5pN \approx \kappa\left\langle \left|x_B\right| \right\rangle$ is constant until it intersects and overlaps with the translational viscous force $F_{\gamma_B,tran}$ of the bead, which is dominant at high frequency.

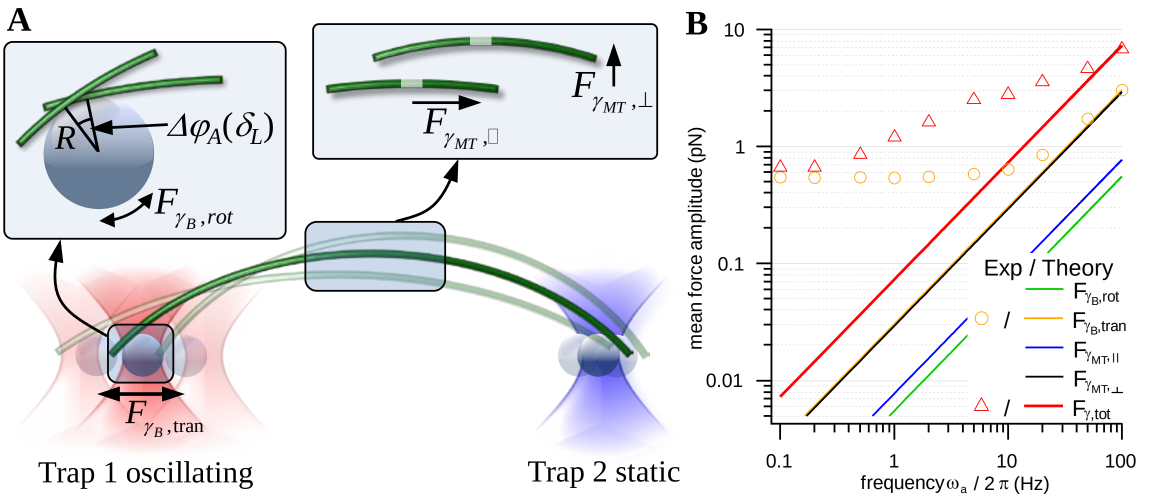

Fig. S5. Estimation of viscous forces. (A) Illustration of experimental situation for a single filament held by two optically trapped beads, one static (trap 2) and one oscillating (trap 1). (B) Frequency dependence of theoretically obtained and experimentally measured force contributions.

For the experimental situation with MT filament, the total force increases continuously but still intersects with the sum of all estimates of the viscous contributions made above at approximately f = 100Hz. This indicates a strong additional contribution from the MT, very likely of elastic nature as we obtained by the micro-rheology analysis for G'.

Contributions of deformation modes to $G'(\omega)$

As stated in the main paper, we estimate to excite N = 3 deformation modes at oscillation frequencies up to f_a = 100Hz. The contributions of each additional mode are illustrated in Fig. S6 where we plotted the theoretical slope of $G'(\omega,N)=\frac{1}{2.16\pi^2}q_1^4 k_B T \ell_p \,\mathrm{Re}\left(\sum_{n=1}^{N}\frac{1}{n^4+i\omega/\omega_1}\right)^{-1}$ for the sum of different deformation modes N = 1, 2, 3 and 10 (solid lines) according to Eq. 5. We normalized the shear modulus $G'(\omega,N)/G'(0,N)$ to obtain a better estimate for the relative contributions. We also plotted the difference $G'(\omega,N)-G'(\omega,N-1)$ (dashed lines) to indicate the influence of a single mode and at which frequency the next mode kicks in.

The first mode, causing a constant plateau, is dominant for low frequencies up to $\omega \approx \omega_1$. Higher deformation modes are excited and result in an increased filament stiffness for frequencies $\omega \geq 3\omega_1 = \omega_{t2} = \omega_t$ (N = 2) and $\omega \geq 20\omega_1 = \omega_{t3}$ (N = 3). Modes higher than $N \geq 4$ are relevant only for frequencies $\omega > 100\omega_1$, which is beyond the experimentally addressed frequency limit in our study.

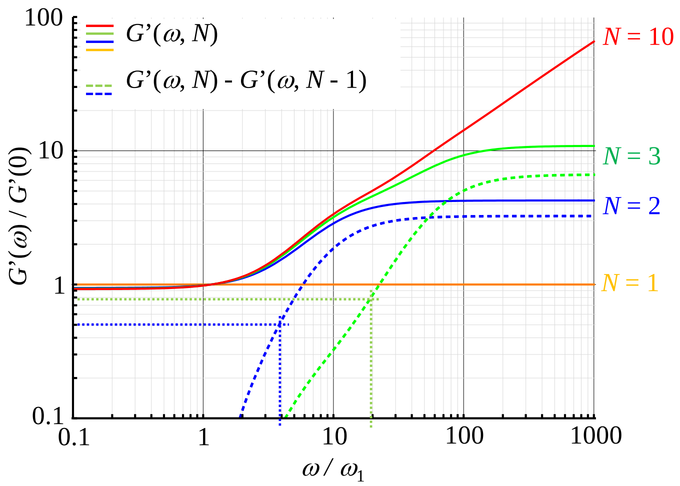

Fig. S6. Theoretical estimate of mode dependence of $G'(\omega)$. The elastic modulus $G'(\omega, N)$ is shown including a different number N of deformation modes n (solid lines) as well as the difference of $G'(\omega, N) - G'(\omega, N-1)$ for different modes (dashed lines).

Viscous components of single filaments

The viscous modulus $G''(\omega)$ of a simple bead in a ideally viscous solution such as water is $G''(\omega) = \gamma_B \cdot \omega / 6\pi R_B = \eta\omega$ (1). Similarly, the viscous component of a rod can be expected to be $G''(\omega) = \gamma_{MT} \cdot \omega / 4\pi L \sim \eta\omega$, i.e., linear with respect to the frequency ω. Indeed, this is what we observe for the viscous modulus of all single filaments as shown in Fig. S7A. The results still depend on the length L_{MT} of the filament due to the logarithmic dependence of $\gamma_{MT} \sim 1 / (\ln(L_{MT} / D) + 0.84)$ on L_{MT} but does not depend on filament stabilization, contrary to $G'(\omega)$ as described in the main paper.

A comparison to the theoretical prediction shown in Fig. S7B reveals that high deformation modes $n \geq 2$ only slightly change this linear relationship and only for relatively high frequencies $\omega > 10\omega_1$, which is roughly the maximum frequency we resolve in our experiments ($\omega_1 \approx 4$Hz typically, see main paper).

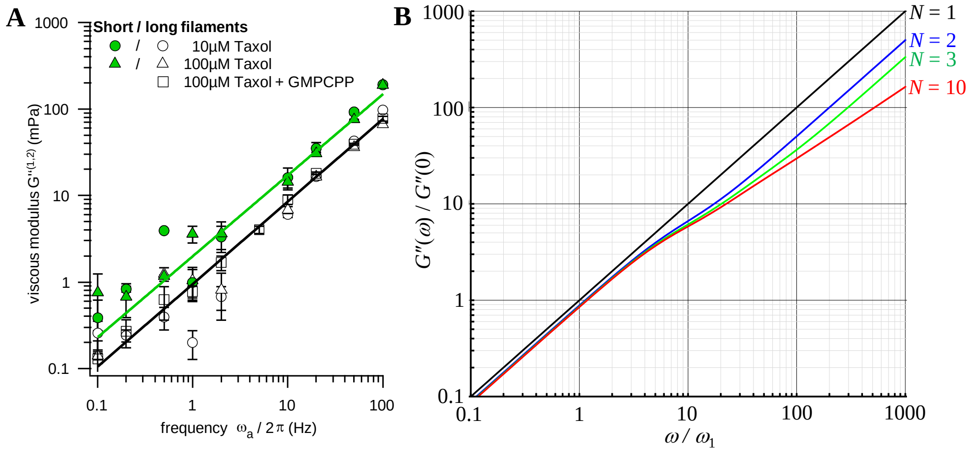

Fig. S7. Viscous components $G''(\omega)$ of single filaments. (A) Experimental results. Solid lines represent linear fits to the data. (B) Theoretical slopes for different number of oscillation modes N.

Viscous components of a linear connection of MTs

Similarly to the viscous components of single MT filaments, the viscous components $G''^{(1,2)}_{\perp,\parallel}$ and $G''^{(1,3)}_{\perp,\parallel}$ of a linear connection of filaments are again linear with respect to frequency ω as shown in Fig. S8 by power law fits with free exponent $p \approx 1$ in all cases.

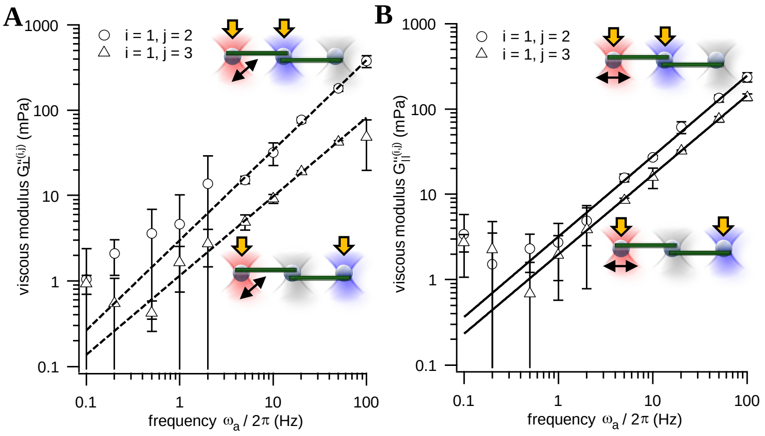

Fig. S8. Viscous components $G''(\omega)$ of a linear connection of filaments. Solid and dashed lines indicate approximately linear fits to the data.

Pre-stress in triangular networks

Free floating filaments are subject to Brownian forces and sometimes bend heavily. This can cause pre-stress during the construction of a network, i.e., the subsequent attachment of a filament to optically trapped beads. This effect becomes more prominent if the number of filaments of a network increases. Fig. S9 shows the elastic modulus *G'* of three different equilateral triangles with a side length of 15µm. For both oscillation directions, the plateau of *G'* is larger for the connection of the first filament 1→2 and smaller for the connection of the second filament 1→3 for the first two triangles, compared to the third triangle where the elastic moduli for both connections are approximately equal. This clearly indicates a pre-stress of the first MT compared to the second filament.

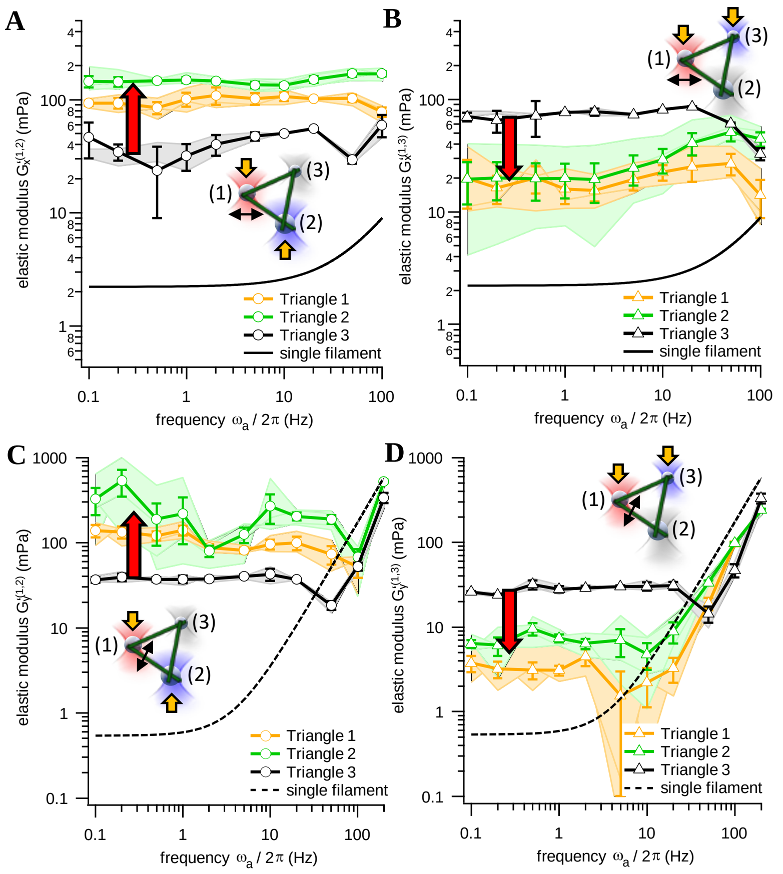

Fig. S9. Pre-stress in an equilateral triangle. (A) and (B) *G*'(*ω*) for a radial oscillation along *x* for the connection 1→2 (A) and 1→3 (B). (C) and (D) *G*'(*ω*) for a tangential oscillation along *y* for the connection 1→2 (C) and 1→3 (D)

Viscous components of triangular networks

Again, we observe a linear relation between the viscous components *G*''(*ω*) of filaments in a triangular networks and the frequency *ω* as shown in Fig. S10 together with power law fits with free exponent $p \approx 1$. For the tangential oscillation along *y*, the viscous component $G''^{(1,2)}$ of the first filament deviates strongly from the expected linear response for the first two triangle constructs, indicating that maybe the connection to either of the beads (1) or (2) was not perfect.

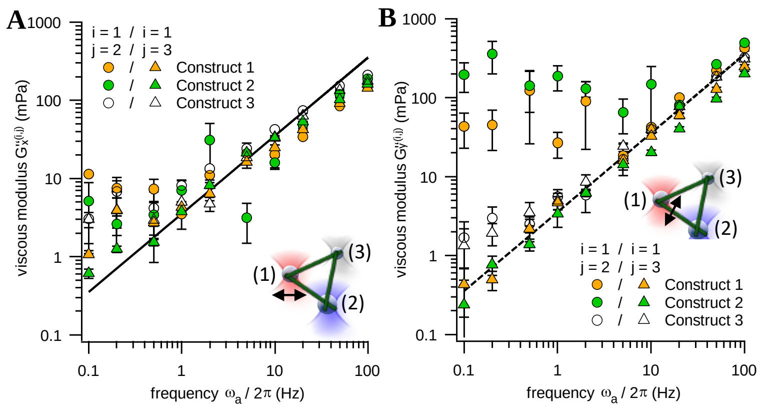

Fig. S10. Viscous components $G''(\omega)$ of a triangular connection of filaments for actor bead displacement along x (left) and along y (right). Solid and dashed lines indicate approximately linear fits to the data.

Comparison of transition frequencies

As described in the main paper, we found that he transition frequency ω_t, separating the constant plateau value of G' for low frequencies from the high frequency rise approximately proportional to $\omega^{1.25}$, depends on filament length, stabilization, polymerization and especially on the geometry of the network. This is summarized in Fig. S11A. The transition frequency is the highest for the triangular network, which is also the stiffest. The difference of filament stabilization is clearly visible for long filaments. There is also a clear difference visible for different oscillation directions of all geometries, where the transition frequency is much smaller for an oscillation lateral to the filament axis, indicating much faster stiffening in this direction.

In order to analyze how well the experimental transition frequencies match the theoretical predictions according to Eq. 3 of the main paper, we plotted the transition frequency as a function of MT contour length as shown in Fig. S11B. Here, we included the length dependence of the MT persistence length $l_p(L,\omega=0)=l_p^\infty/\left(1+\left(l_c/L\right)^2\right)$ according to Pampaloni (18). For the persistence length l_p^∞ of MTs much longer than a critical length l_c = 21µm, we used the values for L = 15µm long MTs obtained in this study to reflect the different stabilizations.

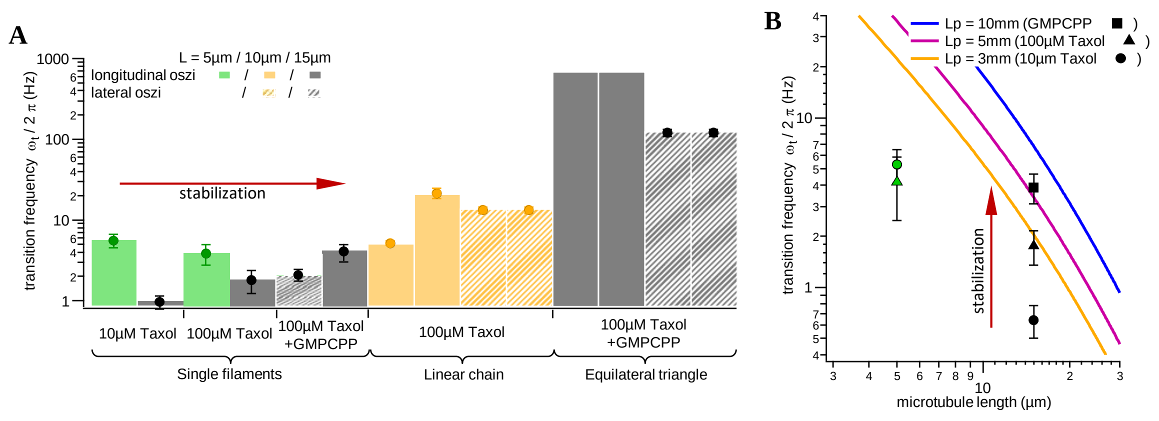

Fig. S11. Transition frequencies for different network geometries, filament stabilizations and polymerizations.

Geometric effects of beads

MT filaments are attached laterally to the anchor beads with a diameter d = 1062nm. This results in a torque on the beads during filament buckling because the optical trapping force acts on their geometric centers. This causes the point of attachment of the filament to a bead to rotate. Hence, the precisely measured distance $\Delta x_L + x_{B1} - x_{B2}$ between both beads does not coincide with the actual projected length p_{MT} of the buckled filament as illustrated in Fig. S12A. For convenience, we assume the symmetric case with $x_{B1} = x_{B2} = x_B$ and $\Delta_1 = \Delta_2 = \Delta_B = R_B \cdot \sin(\varphi) \approx R_B \cdot \varphi$ in the following. Neither the actual compression δ_L given by Eq. (S7) nor the rotation angle $\varphi^2 = 4\delta_L / L_{MT}$ can be measured directly.

$$\delta_L = L_{MT} - p_{MT} = L_{MT} - \left(\Delta x_L + 2x_B - 2\Delta_B\right) \tag{S7}$$

However, both unknowns depend on each other leading to the quadratic relation $\varphi^2 + \frac{8R_B}{L_{MT}}\varphi + 4\left(\frac{\Delta x_L + 2x_B}{L_{MT}} - 1\right) = 0$ and ultimately to an expression for the angle φ which only depends on known or measured quantities given by Eq. (S8).

$$\varphi = \frac{4R_B}{L_{MT}}\left(\sqrt{1 + \frac{L_{MT}}{4R_B^2}(L_{MT} - \Delta x_L - 2x_B)} - 1\right) \tag{S8}$$

This can be substituted in Eq. (S7) to calculate the actual compression δ_L and to plot force compression curves, i.e., the buckling force $F = \kappa_1 \cdot x_{B1} + \kappa_2 \cdot x_{B2}$ versus the compression δ_L as we show for two filaments with different length in Fig. S12C+D. The data shown here were obtained in a quasi-equilibrium where we moved trap 1 in discrete steps of Δx_{L1} = 50nm every Δ_t = 100ms.

In the ideal case, i.e., a perfectly axial application of force on the filament, the MT should not buckle, i.e., $\delta_L(F < F_{crit}) = 0$, until a finite critical buckling force $F_{crit} = \pi^2 EI / L^2_{MT}$ is reached, above which the filament behaves like a spring with spring constant κ_{MT}, i.e., $\delta_L(F > F_{crit}) = (F - F_{crit}) / \kappa_{MT}$ (17). Here, we observe a nearly exponential dependence of the force on the compression for small δ_L < 400nm and a linear dependence for δ_L > 400nm. This is due to the imperfect, lateral application of the force on the filament. We indicated this behavior in Fig. S12C+D by the blue fits with fit function $F(x) = F_{crit}(1 - e^{-\frac{x}{x_0}}) + \kappa_{MT}x$. From this, we determined the critical force $F_{crit}(L_{MT})$ for various filaments of different length as shown in Fig. S12B. As expected from the ideal case, the critical force increases with decreasing filament length, however, not as fast as the ideal $1 / L^2_{MT}$ dependence predicts. This is due to the length dependence of the persistence length $l_p(L_{MT}) = l_p^\infty(1 + \frac{L_c^2}{L_{MT}^2})^{-1}$ (18), which has been used together with $F_{crit}(L_{MT}) = \pi^2 l_p(L_{MT})\, k_B T / L^2_{MT}$ to fit the data. L_c = 3.3µm is the critical filament length above which the persistence length levels to a plateau l_p^∞ = 2.2mm in our case. Pampaloni et al. (18) obtained L_c = 21µm and l_p^∞ = 6mm from their thermal fluctuation data. This deviation is likely a result of different filament polymerization and stabilization. However, we did not consider this geometric effect in our rheology experiments. We expect

this to have only a minor effect on the measured viscoelastic properties G_{MT} of the filaments, but this has to be tested and included into the theory in the future.

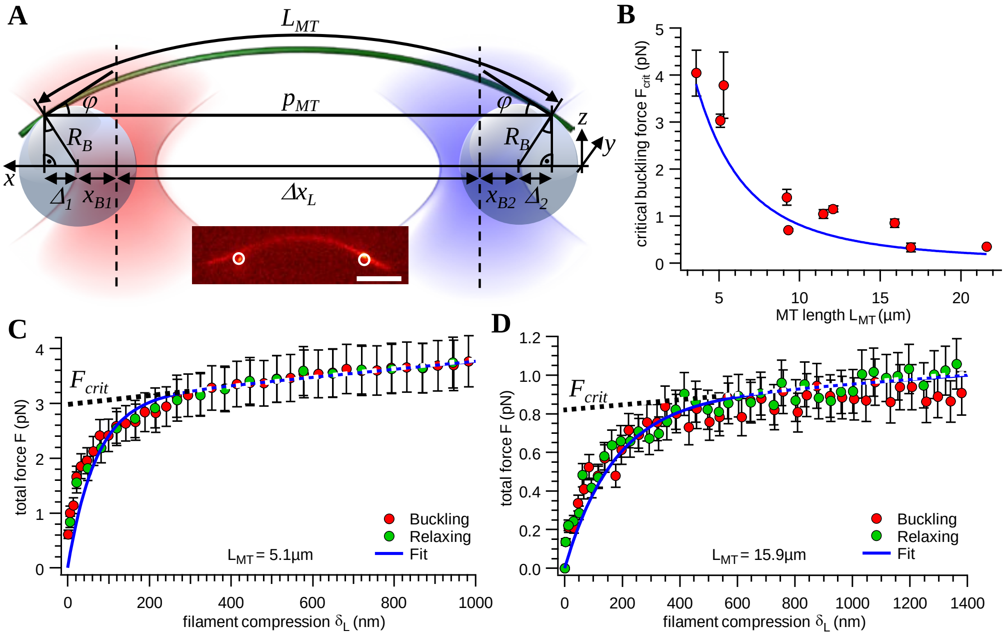

Fig. S12. Geometric effect of filament attachment to the spatially extended anchor beads. (A) Sketch of the experimental situation. (B) Dependence of the critical force $F_{crit}(L_{MT})$ on the filament length L_{MT}. (C) + (D) Plot of the total force $F(\delta_L)$ as a function of filament compression $\delta_L = L_{MT} - p_{MT}$ for a short MT (L_{MT} = 5.1µm, Fig. C) and long (L_{MT} = 15.9µm, Fig. D) MT. Experiments have always been repeated for an increasing buckle and back relaxation to the straight equilibrium position.

Microtubule buckling amplitude

For the integration step in equation (5) of the main manuscript, we need to derive an expression for the buckling amplitude $u_{qn}(\delta_L)$ as a function of the compression $\delta_L(t)$ of the filament with shape $u(x) = \sum_n u_{qn}(\delta_L)\sin(q_n x)$ and an arbitrary bending mode $q_n = \frac{n\pi}{L_{MT}-\delta_L}$.

Considering the invariant arc length $L' = \int \sqrt{1+(\frac{\partial u}{\partial x})^2}\,dx \overset{!}{=} L_{MT}$ of the buckled filament one obtains:

$$L' = \int_0^{L-\delta_L} \sqrt{1+\left(\sum q_n u_{qn}\cos\left(q_n x\right)\right)^2}\,dx \qquad \text{(S9)}$$

Since this square root cannot be solved analytically, we investigate the buckling amplitude u_{qn} as a function of arc length L_n' for single bending modes n, where the ground mode n=1 allows to estimate the maximum possible deflection $u_{q1} > u(x)$.

Substituting $\varphi = q_n x$ leads to

$$L_n' = \int_0^{n\pi} \sqrt{\tfrac{1}{q_n^2} + u_{qn}^2 \cos^2(\varphi)}\, d\varphi = 2n \int_0^{\pi/2} \sqrt{\tfrac{1}{q_n^2} + u_{qn}^2 \cos^2(\varphi)}\, d\varphi \qquad \text{(S10)}$$

Using $\cos^2(\varphi) = 1 - \sin^2(\varphi)$, equation (S10) can be rewritten in the form

$$L_n' = 2n\sqrt{\tfrac{1}{q_n^2} + u_{qn}^2} \int_0^{\pi/2} \sqrt{1 - \frac{u_{qn}^2}{\frac{1}{q_n^2} + u_{qn}^2} \sin^2(\varphi)}\, d\varphi \qquad \text{(S11)}$$

Substituting $u_{qn}' = 2n\sqrt{\tfrac{1}{q_n^2} + u_{qn}^2}$ and $\phi = \frac{u_{qn}}{\sqrt{\frac{1}{q_n^2} + u_{qn}^2}} = 2n\frac{u_{qn}}{u_{qn}'}$ the relation

$$L_{MT} \overset{!}{=} L_n' = u_{qn}' E_{is\phi}\left(\phi(\delta_L)\right) \qquad \text{(S12)}$$

can be deduced. $E_{is\phi}(\phi) = \int_0^{\frac{\pi}{2}} \sqrt{1 - \phi^2 \sin^2(x)}\, dx$ is the complete elliptical integral of the second kind, which cannot be solved analytically. However, an approximation formula $E_{is\phi} = \frac{\pi}{2}\left(1 - \frac{1}{4}\phi^2 - \frac{9}{192}\phi^4 + O(\phi^6)\right)$ can be used (19), leading to a first order approximation $L_{MT} = u_{qn}' \frac{\pi}{2} = n\pi\sqrt{\frac{1}{q_n^2} + u_{qn}^2}$ and ultimately to

$$u_{qn}(\delta_L) = \frac{1}{n\pi}\sqrt{L_{MT}{}^2 - \left(L_{MT} - \delta_L\right)^2} = \frac{1}{n\pi}\sqrt{2L_{MT}\delta_L(t) - \delta_L^2(t)} \qquad \text{(S13)}$$

As shown in Fig. S13 for a microtubule with length L_{MT} = 10 µm, the buckling amplitude u_{qn} increases rapidly with small compressions δ_L < 50 nm and then approximately linearly for larger compressions δ_L > 100 nm. The buckling amplitude decreases with the mode number n. However, the sum of all deformations in a filament is always smaller than the ground mode buckling, i.e. $u(x) < u_{q1}$.

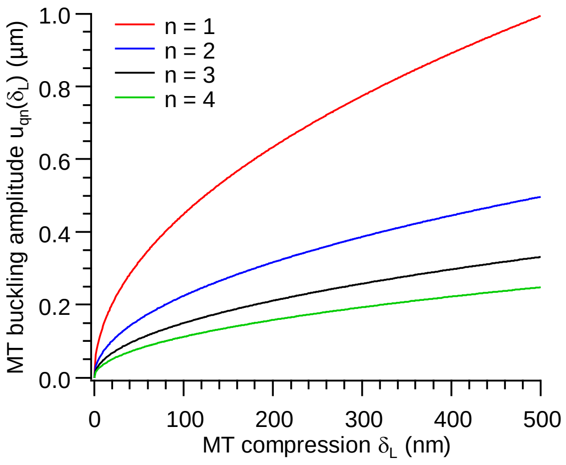

Fig. S13. Buckling amplitude $u_{qn}(\delta_L)$ as a function of filament compression δ_L and mode number n for a filament with length L_{MT} = 10µm.

Linear system theory

Our theoretical description as well as our analysis assume a linear relationship between the microtubule buckling amplitude u_{qn} and the driving force F_D, such that $\tilde{u}_{qn}(\omega) = \alpha_{qn}(\omega)\tilde{F}_D(\omega)$.

In order to test whether the buckling responses of single MTs are linear with force, we analyzed the force dependency $F(\delta_L)$ on a stepwise MT compression by δ_L. Fig. S14 shows that $F(u_{qn})$ increases indeed roughly linearly for not too large buckling amplitudes $u_{qn}(\delta_L)$, in accordance with to Eq. S13.

Linearity: By analyzing the normalized χ^2 value as a function of the number of data points included in a linear fit to the data, we find an approximately linear response up to $u_{qn} \leq 300$ nm for short microtubules (L = 5µm) and $u_{qn} \leq 1.4$µm for long filaments (L = 15µm). This is equivalent to an oscillation amplitude of the laser trap $x_L < 500$nm for short and $x_L < 1200$ nm for long microtubules, respectively. In our rheology experiments, we usually analyze the microtubule response for three different oscillation amplitudes A_a = 200 nm, A_a = 400 nm, and A_a = 600 nm. Hence, linear response is well fulfilled for long microtubules and at least for the two smaller oscillation amplitudes for short filaments. In addition, we always compare the results for different oscillation amplitudes to each other and never observe a significant difference.

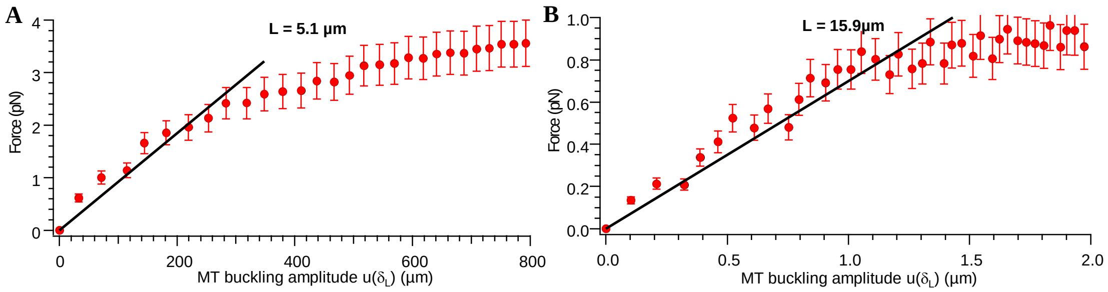

Fig. S 14. Compression force as a function of filament buckling amplitude for a short (A) and long (B) microtubule at $\omega \approx 0$.

Local forces acting along the filament

The curve of a filament deformed in the mode q_n can be described by the vector $\vec{r}(x) = \begin{pmatrix} x \\ u_{qn}\sin(q_n x) \end{pmatrix}$ and has a tangent vector

$$\vec{T}(x) = \frac{d\vec{r}(x)}{dx} \Big/ \left|\frac{d\vec{r}(x)}{dx}\right| = \frac{1}{\sqrt{1+u_{qn}^2 q_n^2 \cos^2(q_n x)}} \cdot \begin{pmatrix} 1 \\ u_{qn} q_n \cos(q_n x) \end{pmatrix}, \qquad \text{(S14)}$$

such that the normal vector is $\vec{N}(x) = \begin{pmatrix} T_y(x) \\ -T_x(x) \end{pmatrix}$. From this, the local elastic force on an infinitesimal section of the filament can be calculated, according to $\vec{f}(x) = EI \cdot \frac{d^4 u(x)}{dx^4} \vec{N}(x) = EI \cdot \frac{u_{qn} \cdot q_n^4 \cdot \sin(q_n x)}{\sqrt{1+u_{qn}^2 q_n^2 \cos^2(q_n x)}} \begin{pmatrix} u_{qn} q_n \cos(q_n x) \\ -1 \end{pmatrix}$, leading to a total force which acts only in y direction

$$\vec{F}_{\kappa,MT}(x) = \int_0^{L-\delta_L} \vec{f}(x)dx = \frac{EI \cdot q_n^3}{u_{qn}} \cdot \left[\begin{pmatrix} -\sqrt{1+u_{qn}^2 q_n^2 \cos^2(q_n x)} \\ \operatorname{asinh}(u_{qn} q_n \cos(q_n x)) \end{pmatrix} \right]_0^{L-\delta_L} = \begin{pmatrix} 0 \\ F_y \end{pmatrix} \qquad \text{(S15)}$$

We like to point out the strong dependence of the local bending force on the third power of the mode number n, meaning that the highest present order always dominates the buckling force.

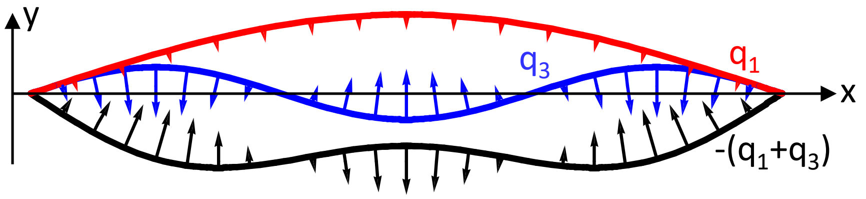

Fig. S 15. Filament deformation for the first and the third mode and the summation of both (flipped vertically). The resulting local force vectors are perpendicular to the filament tangent.

Additional references

1. Atakhorrami M, *et al.* (2006) Correlated fluctuations of microparticles in viscoelastic solutions: quantitative measurement of material properties by microrheology in the presence of optical traps. *Physical Review E* 73(6):061501.
2. Mizuno D, Head D, MacKintosh F, & Schmidt C (2008) Active and passive microrheology in equilibrium and nonequilibrium systems. *Macromolecules* 41(19):7194-7202.
3. Rohrbach A, Tischer C, Neumayer D, Florin EL, & Stelzer EHK (2004) Trapping and tracking a local probe with a photonic force microscope. *Review of Scientific Instruments* 75(6):2197-2210.
4. Svoboda K & Block SM (1994) Biological applications of optical forces. *Annual Review of Biophysics and Biomolecular Structure* 23:247-285.
5. Hawkins TL, Sept D, Mogessie B, Straube A, & Ross JL (2013) Mechanical properties of doubly stabilized microtubule filaments. *Biophysical journal* 104(7):1517-1528.
6. Hawkins T, Mirigian M, Selcuk Yasar M, & Ross JL (2010) Mechanics of microtubules. *Journal of biomechanics* 43(1):23-30.
7. Müller-Reichert T, Chrétien D, Severin F, & Hyman AA (1998) Structural changes at microtubule ends accompanying GTP hydrolysis: information from a slowly hydrolyzable analogue of GTP, guanylyl (α, β) methylenediphosphonate. *Proceedings of the National Academy of Sciences* 95(7):3661-3666.
8. Elie-Caille C, *et al.* (2007) Straight GDP-tubulin protofilaments form in the presence of taxol. *Current Biology* 17(20):1765-1770.
9. Amos LA & Löwe J (1999) How Taxol® stabilises microtubule structure. *Chemistry & biology* 6(3):R65-R69.
10. Nogales E, Wolf SG, Khan IA, Ludueña RF, & Downing KH (1995) Structure of tubulin at 6.5 Å and location of the taxol-binding site. *Nature*.
11. VanBuren V, Cassimeris L, & Odde DJ (2005) Mechanochemical model of microtubule structure and self-assembly kinetics. *Biophysical Journal* 89(5):2911-2926.
12. Ziebert F, Mohrbach H, & Kulić IM (2015) Why Microtubules Run in Circles: Mechanical Hysteresis of the Tubulin Lattice. *Physical Review Letters* 114(14):148101.
13. Ojeda-Lopez MA, *et al.* (2014) Transformation of taxol-stabilized microtubules into inverted tubulin tubules triggered by a tubulin conformation switch. *Nature materials* 13(2):195-203.
14. Maurer SP, Fourniol FJ, Bohner G, Moores CA, & Surrey T (2012) EBs recognize a nucleotide-dependent structural cap at growing microtubule ends. *Cell* 149(2):371-382.
15. Pampaloni F & Florin E-L (2008) Microtubule architecture: inspiration for novel carbon nanotube-based biomimetic materials. *Trends in biotechnology* 26(6):302-310.
16. Tuszyński J, Luchko T, Portet S, & Dixon J (2005) Anisotropic elastic properties of microtubules. *The European Physical Journal E: Soft Matter and Biological Physics* 17(1):29-35.
17. Howard J (2001) *Mechanics of Motor Proteins and the Cytoskeleton*.
18. Pampaloni F, *et al.* (2006) Thermal fluctuations of grafted microtubules provide evidence of a length-dependent persistence length. *Proceedings of the National Academy of Sciences of the United States of America* 103(27):10248-10253.
19. Bronstein IN, *et al.* (2001) *Taschenbuch der Mathematik* (Springer) 5 Ed.
